# Cultivating medical humanistic literacy through immersive case-based practical teaching in undergraduate pathophysiology education

**DOI:** 10.3389/fmed.2026.1861607

**Published:** 2026-06-24

**Authors:** Xueqi Lai, Ying Shen, Ruiheng Zhang, Hongmei Peng, Ying Wang, Yuantao Le, Xianjin Yang, Deling Zhang, Lei Wei

**Affiliations:** 1 Department of Pathophysiology, Wuhan University Taikang Medical School (School of Basic Medical Sciences), Wuhan, Hubei, China; 2 The First Clinical College of Wuhan University, Wuhan, Hubei, China; 3 School of Computer Science, Wuhan University, Wuhan, Hubei, China; 4 School of Marxism, Wuhan University, Wuhan, Hubei, China; 5 The Second Clinical College of Wuhan University, Wuhan, Hubei, China

**Keywords:** digitalized teaching, immersive case-based practical teaching module, medical humanistic literacy, pathophysiology, undergraduate medical education

## Abstract

**Objectives:**

Building on the pathophysiology curriculum, this study aims to explore a novel model for medical humanities education empowered by digital and intelligent technologies. It addresses critical issues, including the lack of humanities-oriented learning contexts and effective pathways for cultivating humanities competence in traditional medical education.

**Methods:**

Utilizing a self-developed digital and intelligent case-based practice platform, the research team integrated medical humanities scenarios to construct an immersive case-based practical teaching module for the pathophysiology course. A total of 80 medical students were randomly assigned to the control group adopting the conventional case discussion teaching module or the experimental group adopting the immersive case-based practical teaching module. A multidimensional evaluation was conducted using a medical humanistic literacy scale, analysis of case discussion class recordings, exam scores, and surveys on extracurricular practice activities.

**Results:**

The experimental group scored significantly higher than the control group in medical humanistic literacy, correct rates on chapters covered in the case discussions, and enthusiasm for extracurricular medical practice activities. Additionally, the experimental group demonstrated an emotional orientation centered on the “patient.”

**Conclusion:**

The immersive case-based practical teaching module in pathophysiology effectively fosters medical humanistic literacy and enhances it synergistically with professional learning. This module contributes to the early cultivation of medical humanistic literacy and advances digital and intelligent educational innovation in medical humanities.

## 1 Introduction

Medical humanism serves as the foundational value of medical practice and is a key pillar in fostering harmonious doctor-patient relationships and enhancing the humanity of medical care. The “Notice on issuing the action plan for enhancing medical humanistic care (2024–2027)” jointly issued by the General Office of the National Health Commission, the General Office of the Ministry of Education, the General Department of the National Administration of Traditional Chinese Medicine, and the General Department of the National Center for Disease Control and Prevention of the People’s Republic of China, explicitly mandates that the cultivation of medical humanistic qualities be integrated throughout the entire process of medical students’ education and professional development (1). However, medical humanities education in China currently lags behind, and the teaching system urgently needs optimization (2). In 2020, the State Council of China issued the “Guiding opinions on accelerating the innovative development of medical education,” which proposed strengthening the cultivation of medical students’ professional ethics and promoting the deep integration of information technology with medical education (3). This provides a new approach of “digital and intelligent empowerment” to overcome the bottlenecks in medical humanities education.

Against this backdrop, the pathophysiology course holds unique value as a required medical course bridging basic and clinical medicine. It aims to investigate the mechanisms and patterns of functional and metabolic changes during the onset and progression of diseases. Its primary mission is to elucidate the nature of diseases and provide theoretical and experimental foundations for developing effective diagnostic, therapeutic, and preventive strategies. The course includes extensive case discussion sessions; the practical nature of case analysis offers a platform for medical humanities education, while the abstract theoretical knowledge provides opportunities for the concrete application of digital and intelligent technologies (4–6). Furthermore, the course is open to second-year medical students across multiple medical specialties, coinciding with a critical stage for the early cultivation of medical humanistic literacy.

Consequently, guided by situated learning (7), experiential learning (8), and self-determination theories (9, 10), this study developed a digitally intelligent immersive case-based practical teaching module for pathophysiology courses. Empowered by digital technologies, this teaching module focuses on cultivating students’ five core medical humanistic literacy dimensions, covering doctor-patient communication, team collaboration, professional emotion, medical reflection, and ethical awareness. By integrating humanistic cultivation with professional learning, it aims to foster students’ comprehensive medical humanistic literacy, improve clinical problem-solving abilities, and shape patient-centered clinical thinking. Distinct from conventional case-based teaching, this innovative module establishes a whole-course system to foster medical humanistic literacy. It promotes the integration of medical humanities education, early professional courses and digital technologies, offering a viable strategy for medical humanities education in the digital age.

## 2 Methods

### 2.1 Study design

This study was designed and reported in accordance with the SQUIRE-EDU (Standards for Quality Improvement Reporting Excellence in Education) guidelines for educational intervention and quality improvement research (11). A randomized parallel-group educational intervention trial was conducted to evaluate the effects of the immersive case-based practical teaching module on medical humanistic literacy, professional knowledge acquisition, and motivation of clinical practice.

### 2.2 Setting

The study was conducted in pathophysiology case discussion courses at Wuhan University, using smart classrooms equipped with a self-developed digital and intelligent case-based practice platform.

### 2.3 Participants and sample

The recruited participants were second-year undergraduate students majoring in clinical medicine or stomatology with equivalent pre-course academic foundations. Inclusion criteria were as follows: (1) voluntary participation in this study with informed consent obtained; (2) full enrollment in the 2025 undergraduate pathophysiology course at Wuhan University; (3) full attendance and completion of the entire teaching intervention; and (4) no prior participation in similar immersive teaching interventions. Exclusion criteria were as follows: (1) incomplete participation in teaching activities; (2) missing questionnaire or assessment data; and (3) withdrawal during the study period.

An a priori sample size calculation was conducted using the non-parametric formula for the Spearman’s rank correlation (12). The required sample size per group was determined as:


$$n=\frac{1}{A\,R\,E}\times \frac{{\left(Z_{1-\alpha /2}+Z_{1-\beta }\right)}^{2}\,\left(1-r^{2}\right)}{2\,r^{2}}$$

where Z_1−α/2_ = 1.96 (α = 0.05, two-tailed), Z_1−β_ = 0.84 (power = 1−β = 0.80), and ARE (Asymptotic Relative Efficiency) = 0.912 for the Spearman’s rank correlation relative to the Pearson correlation under bivariate normality (12). A medium effect size of *r* = 0.35 was adopted (13). Substituting these values yielded a minimum required sample size of approximately 31 participants per group. With an anticipated exclusion rate not exceeding 20%, the target recruitment was set at 37 per group. In practice, 85 participants were enrolled and, after applying the exclusion criteria, 80 valid participants were retained (effective rate, 94.1%), satisfying the a priori power requirement.

### 2.4 Randomization and blinding

Participants were randomly assigned in a 1:1 ratio to the control group (*n* = 40) or experimental group (*n* = 40) using a random number table. The allocation sequence was generated by an independent researcher not involved in participant recruitment, teaching implementation, or outcome assessment. Allocation concealment was ensured using sequentially numbered, sealed, opaque envelopes. Baseline characteristics, including gender and major, were comparable between the two groups (*P* > 0.05). Before intervention initiation, participants and teaching staff were kept unaware of group assignment. Owing to the nature of educational intervention, full blinding of them throughout the teaching process was not feasible. However, personnel responsible for data collection and outcome assessment remained fully blinded to group assignment throughout the study period to minimize assessment bias.

### 2.5 Intervention development and implementation

#### 2.5.1 Development of a case-based practice platform

This study independently developed a case-based practice platform that deconstructs the content of paper-based medical records and constructs multiple clinical practice scenarios based on the clinical diagnosis and treatment process. These scenarios encompass a series of sequential steps, including patient intake, vital sign monitoring, physical examination, auxiliary examinations, diagnosis, and clinical treatment. These steps create an immersive learning scenario through visualized materials that integrate virtual and real elements, including real-world hospital settings, live demonstrations, medical documentation, virtual animations, and ESP wearable devices, thereby enhancing students’ practical learning experience. Students use large touchscreen devices in smart classrooms to interact with the system powered by the DeepSeek large language model. By completing challenge-based tasks and simulated treatment sessions, they progressively unlock the full medical record workflow and receive real-time feedback on their learning progress from the system. The platform is user-friendly, with sessions lasting 30–50 min, and is well-suited for classroom-based practical instruction.

#### 2.5.2 Design of the immersive case-based practical teaching module

This study employs the case-based practice platform as its primary resource, integrating five medical humanities themes—ethics, communication, collaboration, emotions, and reflection—throughout the entire teaching process to establish the immersive case-based practical teaching module. Before class, medical ethics case studies are shared via MOOCs to guide students to independently acquaint themselves with ethical norms, such as Beauchamp and Childress Four Principles of Biomedical Ethics. During class, the case-based practice platform simulates clinical scenarios. Students work in groups of four to six to deepen their understanding of humanistic contexts through doctor-patient role-playing, team challenges, simulated rescues, and group discussions. After class, students review clinical cases and reflect on medical ethical behavior and decision-making through assignments. The digital and intelligent resources employed in this module include the case-based practice platform (equipped with ESP wearable devices), MOOCs, and Chaoxing AI Intelligent Learning Assistant.

#### 2.5.3 Teaching implementation

Students in both the control and experimental groups received six academic hours of case discussion instruction, delivered by instructors from the course team with equivalent qualifications. The case studies used for the discussions were identical in content, covering five chapters from the pathophysiology course, including hypoxia, shock, and acute renal failure. The control group employed a conventional case discussion teaching module, while the experimental group adopted the immersive case-based practical teaching module; specific details and differences are shown in Table 1. Questionnaire surveys and objective assessments were conducted upon course completion.

**TABLE 1:** Comparison of case discussion teaching module between two groups (*n* = 40/group).

Group	Control group	Experimental group
Teaching module	Conventional case discussion teaching module	Immersive case-based practical teaching module
Before class	Preview case content	Preview case content and require students to acquaint themselves with ethical norms
During class	(1) Cases presented in text description (2) Paper cases distributed to each discussion group (4–6 students/group); students explore disease mechanisms and treatment decisions, prepare mind maps, PPTs, etc., for oral presentation.	(1) The case-based practice platform simulate authentic clinical scenarios (2) Student teams (4–6 students/group) unlock the full case process through challenge-based tasks and simulate clinical treatment. Students perform doctor-patient role-play throughout the process to experience humanistic communication and potential ethical conflicts in medical practice. (3) Student teams discuss disease mechanisms and treatment decisions, prepare mind maps, PPTs, etc., for oral presentation.
After class	Reflect on the case mechanism in the form of written assignments	Review and reflect on case mechanisms, medical ethical decision-making, the importance of doctor-patient communication, and strategies for resolving communication conflicts in the form of written assignments.

### 2.6 Outcome measures

Scores of the Medical Humanistic Literacy Assessment Scale were defined as the primary and confirmatory outcome, while remaining indicators served as secondary and exploratory outcomes.

#### 2.6.1 Scores on the Medical Humanistic Literacy Assessment Scale

Drawing on established scales from the literature spanning 2000–2025 (14–16), the five-dimensional Medical Humanistic Literacy Assessment Scale was developed and synthesized, as shown in Supplementary File 1.

For construct development, five core dimensions were defined according to medical humanities education theory and clinical practice requirements: doctor-patient communication, team collaboration, professional emotion, medical reflection, and ethical awareness. Items were generated, screened, and revised through expert consultation to ensure content validity and representativeness. For pilot testing, the scale was administered to 30 medical students not included in the formal study. Ambiguous or inappropriate items were revised or deleted based on participant feedback.

For external validation, expert appraisal and statistical validation were performed. Expert appraisal was conducted by specialists in medical education and medical ethics to verify content validity. Statistical validation included reliability testing (Cronbach’s α) and construct validity analysis (KMO test and Bartlett’s test of sphericity).

The final scale comprises 27 items and utilizes a five-point Likert scale, with scores ranging from 1 (strongly disagree) to 5 (strongly agree). Psychometric testing yielded Cronbach’s α of 0.950, KMO of 0.855, and a significant result in Bartlett’s test of sphericity (*P* < 0.001), indicating that the scale was valid and reliable for assessing medical students’ medical humanistic literacy. Data were collected via SoJump platform.

#### 2.6.2 Scores on the Sense of Gain Scale for the Immersive Case-based Practical Teaching Module

Based on the maturity scale proposed by Ko et al. (17), the Sense of Gain Scale for the Immersive Case-based Practical Teaching Module was developed, as shown in Supplementary File 2. A five-point Likert scale was employed, with scores ranging from 1 (strongly disagree) to 5 (strongly agree). Data were collected via SoJump platform.

#### 2.6.3 High-frequency words and sentiment scores in immersive case-based practical teaching class recordings

Record the audio from the immersive case-based practical teaching classes of the experimental group, transcribe the recordings, and use the WeiCiYun website^1^ to analyze word frequency, semantic networks, and sentiment scores.

#### 2.6.4 Exam accuracy rate

Type A2 questions (case-based multiple-choice questions) were used to assess students’ mastery and application of knowledge in the taught pathophysiology chapters. The correct answer rates in chapters not covered in the case discussions and chapters covered in the case discussions were compared between two groups. The correct rate was calculated as follows:


Correct⁢rate=(number⁢of⁢correct⁢responses÷total⁢responses)×100%

#### 2.6.5 Survey on extracurricular medical practice

A questionnaire survey was conducted to investigate the types, frequency of students’ participation in extracurricular medical practice during the 2-month vacation after the course, as well as their willingness to participate in such practice in the following year. This questionnaire is shown in Supplementary File 3.

### 2.7 Statistical analysis

Data analysis was performed using SPSS 27.0 and GraphPad Prism 7. Descriptive statistics were conducted for medical humanistic literacy scores, high-frequency discussion words, exam accuracy rates, and extracurricular medical practice frequency and willingness scores. Skewed distribution data were presented as median and interquartile range [M (Q1, Q3)] and compared between groups using the Mann-Whitney U test. Categorical data were expressed as frequencies and percentages [*n* (%)] and compared using the χ^2^ test. Spearman’s rank correlation (*r*_*s*_) was adopted to analyze the relationship between sense of gain from the immersive case-based practical teaching module and medical humanistic literacy in the experimental group. Effect sizes were calculated, with 0.1, 0.3, and 0.5 defined as small, medium, and large levels. *P* < 0.05 was considered statistically significant.

### 2.8 Ethical considerations

This study protocol was reviewed and approved by the Wuhan University Committee on Life and Medical Ethics (No. WHU-LFMD-IRB2025074), and informed consent was obtained from all participants. All methods were performed in accordance with the Declaration of Helsinki.

## 3 Results

### 3.1 Scores on the Medical Humanistic Literacy Assessment Scale

As defined in the study methods, scores on the Medical Humanistic Literacy Assessment Scale served as the primary and confirmatory outcome. the scale consisted of five dimensions. The overall score in the experimental group was significantly higher than that in the control group (*r* = 0.254, *P* = 0.023). Specifically, scores for four dimensions—doctor-patient communication, team collaboration, professional emotion, and medical reflection—were significantly improved (*P* < 0.05), whereas the score for ethical awareness was higher but not statistically significant (*P* > 0.05). Further analysis revealed that the experimental group scored significantly higher on items regarding communication willingness, professional value, medical reflection awareness, and medical reflection habits (*P* < 0.05), whereas no significant differences were found in communication skills and medical reflection competency (*P* > 0.05), as shown in Table 2.

**TABLE 2:** Scores on the Medical Humanistic Literacy Assessment Scale for two groups of students [M (Q1, Q3), *n* = 40/group].

Dimension	Control group	Experimental group	U	Z	*r*	*P*
Overall score	3.96 (3.61, 4.22)	4.04 (3.87, 4.75)	1036.0	2.273	0.254	0.023
Doctor-patient communication	4.00 (3.71, 4.42)	4.17 (4.00, 4.67)	1044.0	2.372	0.265	0.018
Communication willingness	4.00 (3.67, 4.25)	4.17 (4.00, 4.92)	1033.5	2.342	0.262	0.019
Communication skills	4.00 (3.67, 4.58)	4,00 (4.00, 4.58)	975.5	1.737	0.194	0.082
Team collaboration	4.00 (3.67, 4.58)	4.17 (4.00, 5.00)	1017.5	2.181	0.244	0.029
Professional emotion	3.88 (3.50, 4.00)	4.00 (3.75, 4.50)	1086.0	2.821	0.315	0.005
Professional value	3.50 (3.13, 4.00)	4.00 (3.50, 4.88)	1105.0	3.045	0.340	0.002
Empathy	4.00 (3.50, 4.00)	4.00 (4.00, 4.88)	991.5	1.961	0.219	0.050
Medical reflection	3.95 (3.60, 4.28)	4.00 (3.93, 4.58)	1034.0	2.275	0.254	0.023
Medical reflection awareness	3.83 (3.67, 4.25)	4.00 (3.67, 4.67)	1033.5	2.303	0.257	0.021
Medical reflection habits	4.00 (3.50, 4.44)	4.00 (4.00, 4.75)	1073.5	2.706	0.303	0.007
Medical reflection competency	4.00 (3.67, 4.25)	4.00 (4.00, 4.67)	926.5	1.286	0.144	0.198
Ethical awareness	4.00 (3.75, 4.25)	4.00 (4.00, 4.50)	954.0	1.514	0.169	0.130

For Mann–Whitney U tests, effect size *r* was computed as $r\,Z/\sqrt{N}$, where Z is the standardized test statistic, and N is the total sample size. *r* values of 0.1, 0.3, and 0.5 represent small, medium, and large effect sizes, respectively.

**FIGURE 1:**
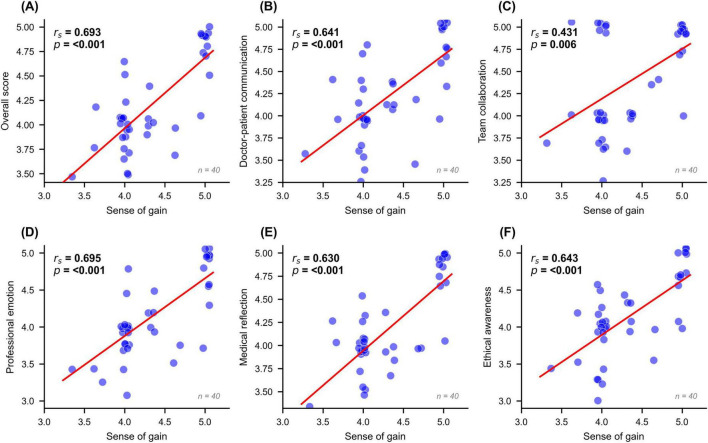
Spearman;s rank correlations between scores on the Sense of Gain Scale for the Immersive Case-based Practical Teaching Module and overall scores **(A)** as well as five dimensional subscores **(B–F)** of the Medical Humanistic Literacy Assessment Scale (experimental group, *n* = 40). The correlation coefficient *r*_*s*_ was adopted as the effect size index; absolute *r*_*s*_ values of 0.1, 0.3, and 0.5 were defined as small, medium, and large effect sizes, respectively.

To further evaluate the effect of the immersive case-based practical teaching module in improving medical humanistic literacy, the experimental group’s sense of gain from the module was assessed using the Sense of Gain Scale for the Immersive Case-based Practical Teaching Module (overall score = 5 points). Scores ranged from 3.3 to 5, reflecting a generally high level of students’ sense of gain from this teaching module. The relationship between sense of gain and medical humanistic literacy was examined using Spearman’s rank correlation. Significant positive correlations were observed between sense of gain and all dimensions of the Medical Humanistic Literacy Assessment Scale, as shown in Figure 1. A strong correlation with a large effect size was identified between sense of gain and the overall score (*r_*s*_* = 0.693, *P* < 0.001). Large effect sizes were also observed in doctor-patient communication (*r_*s*_* = 0.641, *P* < 0.001), professional emotion (*r_*s*_* = 0.695, *P* < 0.001), medical reflection (*r_*s*_* = 0.630, *P* < 0.001), and ethical awareness (*r_*s*_* = 0.643, *P* < 0.001). A moderate correlation existed between sense of gain and team collaboration (*r*_*s*_ = 0.431, *P* = 0.006).

### 3.2 High-frequency words and sentiment scores in case discussion class recordings

**FIGURE 2:**
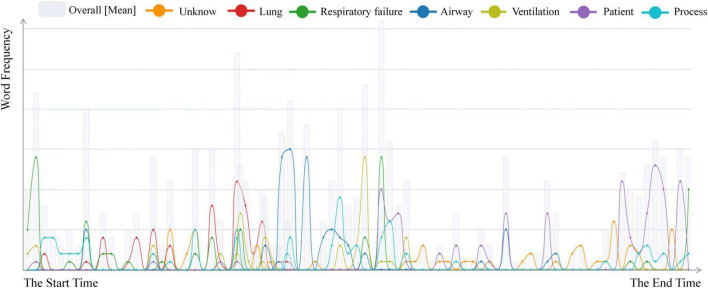
Time rhythm distribution curve of high-frequency keywords in case discussions. The legend illustrates the dynamic changes in the frequency of high-frequency keywords (distinguished by color) throughout the entire case discussion class.

**FIGURE 3:**
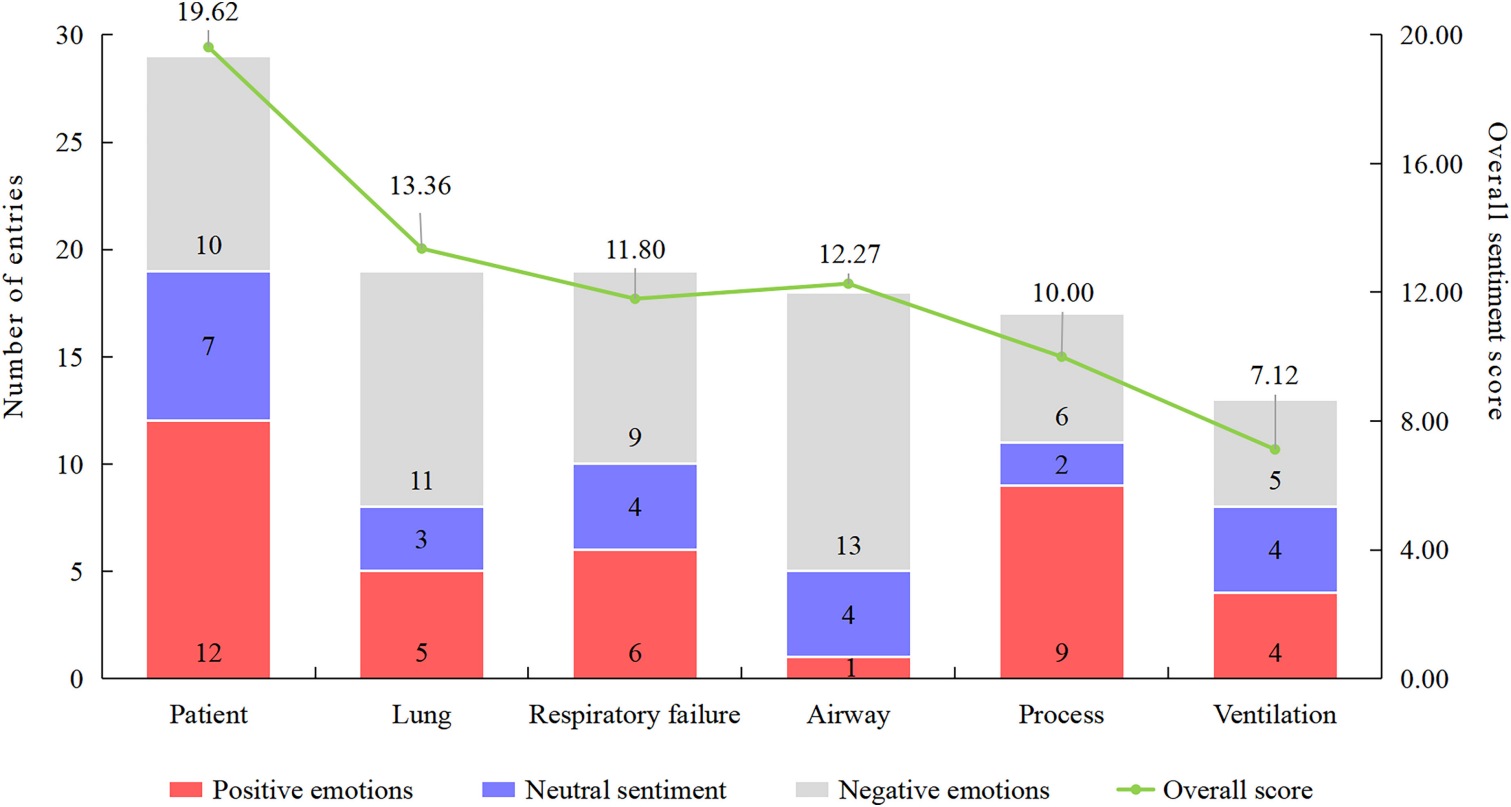
Sentiment scores and number of entries for high-frequency keywords in case discussions. The horizontal axis represents high-frequency keywords. The left vertical axis shows the number of entries corresponding to each keyword, and the right vertical axis represents the overall sentiment score of each keyword. Red, blue, and gray indicate positive, neutral, and negative sentiment entries, respectively, with the green line chart displaying the overall sentiment score.

To explore the discussion themes and emotional changes of students during immersive case discussion class, sentiment analysis was conducted on the full recording of the experimental group’s case discussion class via the Weiciyun website. Taking cases related to the chapters of Hypoxia and Respiratory Failure as examples, in the early stage of class, high-frequency words were dominated by disease-related terms involved in the cases (e.g., “airway,” “respiratory failure”), which featured a high occurrence frequency and large fluctuations. With the progression of the discussion class, the word frequency of “patient” rose gradually and remained at a high level in the later stage, while that of disease-related terms relatively decreased or tended to be stable, as shown in Figure 2. Sentiment scores indicated that “patient” accounted for the highest proportion among high-frequency words (29 entries), with 12, 7, and 10 entries corresponding to positive, neutral and negative sentiment states, respectively. Meanwhile, “patient” also had the highest positive sentiment score (19.62 points), demonstrating a significant positive emotional tendency among students in discussions related to “patient,” as shown in Figure 3.

### 3.3 Exam accuracy rate

To explore the impact of the immersive case-based practical teaching module on learning outcomes, type A2 questions (case-based multiple-choice questions) were used to assess the knowledge application and clinical analysis abilities of the two groups. The results showed that there was no significant difference in the correct rate of type A2 questions between the two groups for chapters not covered in the case discussions (χ^2^ = 0.334, Cramer’s V = 0.037, *P* = 0.563), indicating that two groups of students had consistent learning foundations. In contrast, the experimental group had a significantly higher correct rate for chapters covered in the case discussions (χ^2^ = 4.251, Cramer’s V = 0.133, *P* = 0.039), as shown in Table 3. This suggests that the immersive case-based practical teaching module is conducive to improving students’ professional learning outcomes and promoting the coordinated improvement of medical humanistic literacy and professional learning.

**TABLE 3:** Correct answer rate for type A2 questions in the taught pathophysiology chapters [*n* (%), total *n* = 240].

Classification	Control group	Experimental group	Total	χ^2^	Cramer’s V	*P*
Chapters not covered in the case discussions						
Number of correct responses	85 (70.8%)	89 (74.2%)	174 (72.5%)	0.334	0.037	0.563
Number of incorrect responses	35 (29.2%)	31 (25.8%)	66 (27.5%)			
Total	120	120	240			
Chapters covered in the case discussions						
Number of correct responses	98 (81.7%)	109 (90.8%)	207 (86.3%)	4.251	0.133	0.039
Number of incorrect responses	22 (18.3%)	11 (9.2%)	33 (13.8%)			
Total	120	120	240			

For χ^2^ tests, Cramer’s V was computed as $V=\sqrt{\chi^{2}/N}$, where N is the total sample size. Cramer’s V values of 0.1, 0.3, and 0.5 represent small, medium, and large effect sizes, respectively.

### 3.4 Extracurricular medical practice participation frequency and willingness

**FIGURE 4:**
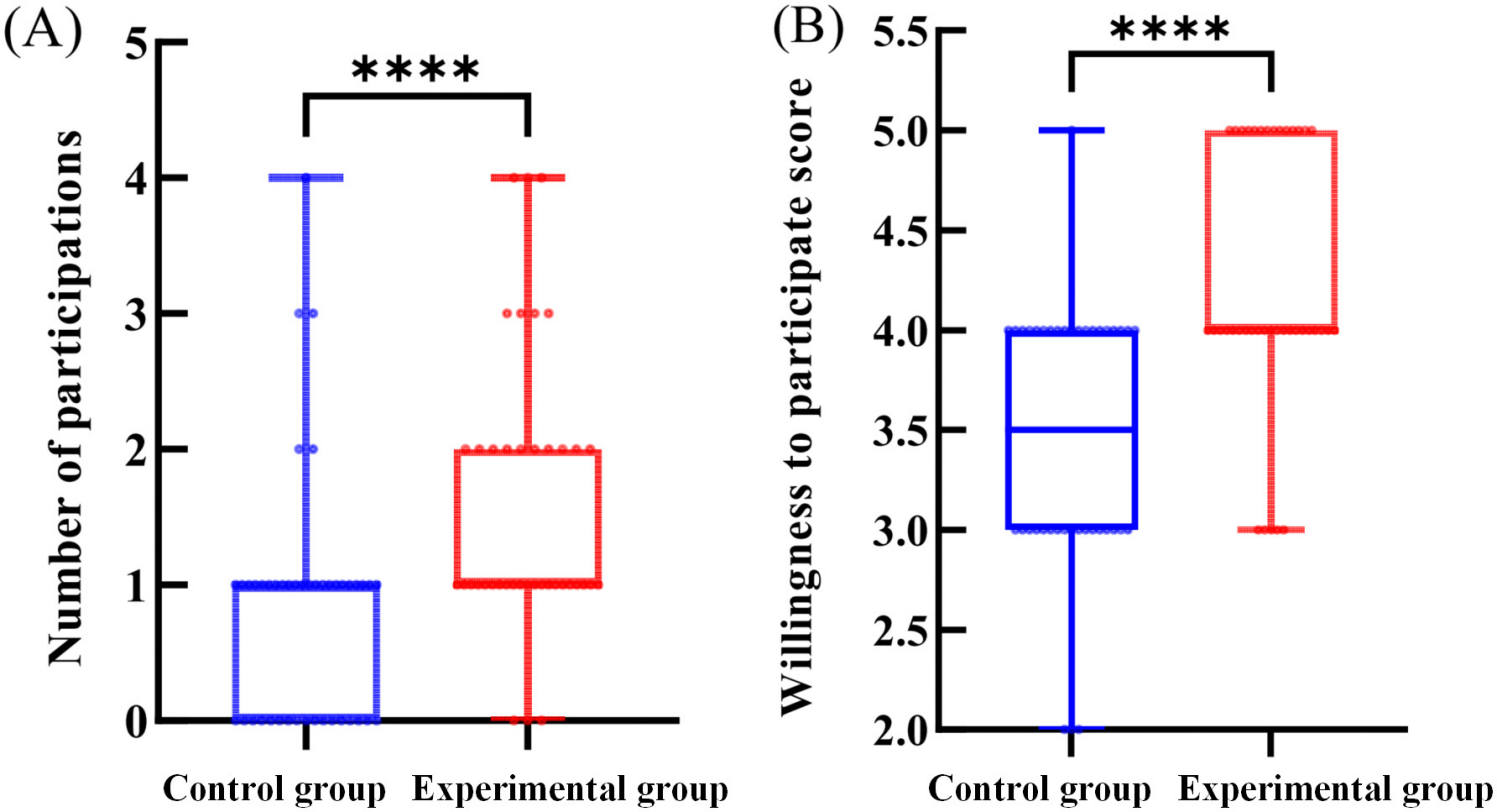
Comparison of extracurricular medical practice participation frequency and willingness between the control and experimental groups. **(A)** Shows the frequency of extracurricular medical practice participation, while **(B)** presents the willingness-to-participate scores. Data are displayed as boxplots with median and interquartile range [M (Q1, Q3)]. Individual data points are overlaid: blue dots for the control group, red dots for the experimental group. The horizontal line within each box represents the median (50th percentile); the box spans the interquartile range (IQR, 25th to 75th percentile); and the error bars extend to the minimum and maximum values. Between-group comparisons were performed using the Mann-Whitney U test. Sample size: *n* = 40 per group. *⁣*⁣**P < 0.0001.

To investigate whether internalized medical humanistic literacy can promote the externalization of professional behavior, a survey on extracurricular medical practice revealed that the experimental group exhibited significantly higher average participation frequency and willingness to participate in future activities than the control group (*P* < 0.0001), as shown in Figure 4. The specific types of extracurricular medical practice activities are presented in Table 4. The experimental group participated more frequently in all types of activities than the control group, among which internship/clerkship and voluntary service showed the highest frequencies.

**TABLE 4:** Types and participation frequency of various extracurricular medical practice activities (*n* = 40/group).

Types of extracurricular activities	Number of participations	
	Control group	Experimental group
Internship/traineeship	13	15
Volunteer service	8	15
Scientific research	11	12
Social practice	4	12
Innovation competition	2	11
Overseas study tours	0	2
Total	38	67

## 4 Discussion

Medical humanities education is the critical approach to cultivating medical humanistic literacy. In recent years, the Chinese government and universities have continuously promoted medical humanities education. However, medical cases that provoke negative public opinion continue to frequently appear in the public eye. This situation reflects, to some extent, deep-rooted deficiencies in medical humanities education within the traditional medical education model. Currently, medical humanities education worldwide faces a significant challenge: the insufficient integration of humanistic literacy training with professional courses, clinical practice, and digital intelligence technologies during early medical education, along with a lack of situational experiential carriers and systematic implementation pathways. As a result, it is difficult for medical students to effectively cultivate their humanistic literacy and translate them into professional practice (2, 4, 5, 18–20). This study integrated medical humanities education into early professional medical courses and established a digital intelligence-enabled case-based practical teaching module to create an immersive medical humanities environment. A synergistic innovation pathway of “technology–module–evaluation” was formed, which precisely addresses the practical pain points of medical humanities education. It successfully overcomes the difficulty of implementing effective medical humanities education for junior medical students due to their lack of clinical experience, and achieves the integrated development of medical humanistic literacy cultivation and professional academic growth.

The immersive case-based practical teaching module in pathophysiology course effectively fosters medical humanistic literacy among medical students. The results of this study showed that the overall score of the Medical Humanistic Literacy Assessment Scale in the experimental group was significantly higher than that in the control group. Within the experimental group, students with a higher sense of gain from the practical teaching module achieved higher scores in all dimensions of medical humanistic literacy, which fully confirms the effectiveness of this module in improving medical humanistic literacy. Recordings-based sentiment analysis further demonstrated that the module enhanced students’ awareness of patient-centered humanistic care.

Both situated learning theory and experiential learning theory emphasize that learning at the attitudinal and value levels is situation-dependent and experience-dependent, requiring internalization within concrete practical fields (7, 8). Research on medical humanities education also indicates that medical humanistic literacy is not abstract moral preaching, but a practical competency characterized by patient-centeredness, empathy, professional responsibility, and ethical judgment, Its cultivation must rely on real clinical problems and situational experiences rather than isolated knowledge instruction (19, 21–23). Accordingly, the effectiveness of this study can be attributed to the following measures. First, the self-developed case-based practice platform dynamically simulates real clinical scenarios through progressive disease courses. Combined with multi-role doctor–patient simulations and team-based tasks, it enhances students’ humanistic communication experiences, promotes the formation of empathetic clinical thinking, breaks through the disease-centered cognitive framework, and facilitates the emotional and practical shift from focusing on “disease” to focusing on “patient.” This reflects the unique value of situated and experiential learning in shaping professional attitudes and fostering behavioral transformation (7, 8). Second, the immersive case-based practical teaching module, which integrates five medical humanistic literacy scenarios—ethics, communication, collaboration, emotion, and reflection—was designed based on experiential learning theory. It promotes deep learning through the “experience–reflection–abstraction–application” cycle (8), forming a dynamic and integrated learning loop that provides an effective pathway for awakening and internalizing medical humanistic literacy.

A detailed analysis of the effects of the immersive case-based practical teaching module on fostering various dimensions of students’ medical humanistic literacy revealed significant improvements in doctor–patient communication, team collaboration, professional emotion, and medical reflection. However, no statistically significant change was observed in ethical awareness. According to the principles of stage-based progression and gradual internalization proposed by moral development theory, the formation and internalization of advanced ethical cognition require repeated exposure to complex moral dilemmas and in-depth reflection (24, 25). Given the relatively short intervention period in this study, the complexity and depth of ethical scenarios provided by the cases were limited, making it difficult to induce significant improvements in ethical awareness. In addition, the results of the scale measuring doctor–patient communication and medical reflection showed significant improvements in attitudes but no notable advances in actual competence. This suggests that awakening awareness of communication and reflection is relatively easy, whereas the construction of practical skills is slower and relies more on repeated cycles of “practice–feedback–revision” and more complex real clinical contexts (7, 8, 23). Therefore, future studies will further optimize simulated case scenarios, increase training intensity and duration, and refine the design of ethical dilemmas.

In this study, the immersive case-based practical teaching module not only enhanced medical humanistic literacy but also significantly improved examination performance and enthusiasm for professional activities. This indicates that the module not only boosts short-term learning outcomes but also fosters long-term, stable motivation for professional practice. The simulated clinical environment and immersive experience effectively stimulate students’ interest in learning, facilitate the translation of abstract disease knowledge into clinical applications, promote the internalization and application of professional knowledge, and enhance learning efficacy (7, 8). More importantly, the internalization of medical humanistic literacy can serve as an intrinsic motivator for learning and professional behavior. Consistent with self-determination theory, immersive case-based practical teaching addresses students’ needs for autonomy, competence, and belonging, helping them develop a stable professional value identity and intrinsic motivation for learning. This approach shifts students from passive learning to active professional exploration driven by humanistic beliefs (9, 10). Consequently, the immersive case-based practical teaching module not only enhances medical humanistic literacy but also positively influences academic performance and professional engagement through the internalization of humanistic values, thereby fostering mutual reinforcement and synergistic improvement between the cultivation of humanistic literacy and professional learning.

Furthermore, this study developed a systematic evaluation framework for medical humanities education, incorporating a refined Medical Humanistic Literacy Assessment Scale, recording analysis of case-based discussion class, examinations, and surveys of medical practice behaviors. It transformed the evaluation of medical humanistic literacy from a subjective, one-dimensional approach into an objective, multidimensional, and quantifiable indicators, enabling precise feedback on educational outcomes and facilitating the scientific transition of medical humanities education from “empirical judgment” to “evidence-based evaluation.”

## 5 Limitations

While this study provides valuable insights into cultivating medical humanistic literacy, several limitations should be acknowledged. First, although teachers with identical professional qualifications were assigned to the two groups to minimize experimental interference, individual differences in teaching styles and classroom interactions may introduce unavoidable subtle teaching bias. Second, this single-center study was conducted at a single medical university using a self-developed browser-based case practice platform. Despite the platform’s convenient deployment and good transferability, the single-site sample limits the generalizability of the findings to other student populations and educational settings. Third, the short intervention duration of the immersive case-based practical teaching module may limit improvements in advanced competencies such as ethical awareness and practical skills, and the long-term effects remain unclear. Furthermore, the ethical dilemmas scenarios in the module require further enrichment.

Future research will address the above limitations. Standardized teacher training will be implemented to unify teaching norms and eliminate individual teaching bias. Multi-center studies with diverse student samples will be conducted to enhance the external validity and generalizability of the results, and the self-developed platform will be optimized for better compatibility and usability to promote broader application. Extended intervention cycles and long-term follow-up will be carried out to validate the sustainable effects of this teaching model on humanistic literacy cultivation. Upgraded digital technologies and sophisticated clinical ethical scenarios will be incorporated into the module to further develop students’ advanced humanistic competencies. Additionally, standardized supplementary teaching protocols for the control group will be designed to ensure educational equity and uphold research ethics.

## 6 Conclusion

In conclusion, the self-developed immersive case-based practical teaching module serves as an effective educational strategy for advancing medical humanities education. It facilitates early cultivation of medical students’ humanistic awareness, features convenient implementation and measurable outcome evaluation, and offers a feasible reference and practical insight for the digital reform of medical humanities teaching.

## Data Availability

The original contributions presented in this study are included in this article/Supplementary material, further inquiries can be directed to the corresponding authors.

## Footnotes

1
https://www.weiciyun.com/
