# Supplementary material for: Cultivating medical humanistic literacy through immersive case-based practical teaching in undergraduate pathophysiology education
Source: Front Med (Lausanne). 2026 Jun 24;13:1861607. doi: 10.3389/fmed.2026.1861607 (PMC13341589; doi:10.3389/fmed.2026.1861607)
Supplement: Supplementary file 1 [file Supplementary_file_1.docx]

**Medical Humanistic Literacy Assessment Scale**

Dear classmates,

This survey aims to investigate the current status of medical humanistic literacy and course learning experience among medical students. The results will be used for teaching research and curriculum optimization. The questionnaire is anonymous, and all data will only be used for overall statistical analysis. We strictly abide by research ethics to ensure the confidentiality of personal information.

There are no right or wrong answers. Please answer truthfully based on your real feelings and actual situation. This survey is only for teaching research purposes and has nothing to do with your course scores or assessment.

Sincerely thank you for your support and cooperation!

Pathophysiology Course Team

**1.Doctor-patient communication**

①Communication willingness

Please read the following statements carefully and choose the degree of agreement based on your true thoughts: [Matrix Single Choice]

|  | **Strongly Disagree** | **Disagree** | **Neutral** | **Agree** | **Strongly Agree** |
| --- | --- | --- | --- | --- | --- |
| I am willing to take the initiative to communicate with patients and their families. | ○ | ○ | ○ | ○ | ○ |
| I can take the initiative to build a trusting doctor-patient relationship with patients and their families. | ○ | ○ | ○ | ○ | ○ |
| I prioritize the emotions and psychological state of patients and their families during communication. | ○ | ○ | ○ | ○ | ○ |

②Communication skills

Please read the following statements carefully and choose the degree of agreement based on your true thoughts:

|  | **Strongly Disagree** | **Disagree** | **Neutral** | **Agree** | **Strongly Agree** |
| --- | --- | --- | --- | --- | --- |
| I tend to explain medical conditions to patients or their families in plain language. | ○ | ○ | ○ | ○ | ○ |
| I can listen effectively and accurately restate the core concerns of patients. | ○ | ○ | ○ | ○ | ○ |
| I can balance factual information and emotional support when delivering bad news. | ○ | ○ | ○ | ○ | ○ |

**2. Team collaboration**

Please read the following statements carefully and choose the degree of agreement based on your true thoughts: [Matrix Single Choice]

|  | **Strongly Disagree** | **Disagree** | **Neutral** | **Agree** | **Strongly Agree** |
| --- | --- | --- | --- | --- | --- |
| In group study or activities, I can communicate proactively with team members to ensure efficient information sharing. | ○ | ○ | ○ | ○ | ○ |
| I can reasonably assign team tasks and give full play to each member’s strengths. | ○ | ○ | ○ | ○ | ○ |
| When facing disagreements, I prioritize reaching a consensus rather than insisting on my own opinions. | ○ | ○ | ○ | ○ | ○ |

1. **Professional emotion**

①Professional value

Please read the following statements carefully and choose the degree of agreement based on your true thoughts: [Matrix Single Choice]

|  | **Strongly Disagree** | **Disagree** | **Neutral** | **Agree** | **Strongly Agree** |
| --- | --- | --- | --- | --- | --- |
| I believe that safeguarding the vital interests of patients is more important than pursuing personal achievement | ○ | ○ | ○ | ○ | ○ |
| I am willing to sacrifice my personal rest time to meet the reasonable medical needs of patients. | ○ | ○ | ○ | ○ | ○ |

②Empathy

Please read the following statements carefully and choose the degree of agreement based on your true thoughts:

|  | **Strongly Disagree** | **Disagree** | **Neutral** | **Agree** | **Strongly Agree** |
| --- | --- | --- | --- | --- | --- |
| I can sensitively perceive the unexpressed worries and needs of patients. | ○ | ○ | ○ | ○ | ○ |
| After thinking from the perspective of patients or their families, I can better understand the difficulties of both doctors and patients in future practice. | ○ | ○ | ○ | ○ | ○ |

1. **Medical reflection**

①Medical reflection awareness

Please read the following statements carefully and choose the degree of agreement based on your true thoughts: [Matrix Single Choice]

|  | **Strongly Disagree** | **Disagree** | **Neutral** | **Agree** | **Strongly Agree** |
| --- | --- | --- | --- | --- | --- |
| I can promptly identify key links that may lead to medical errors. | ○ | ○ | ○ | ○ | ○ |
| For medical errors that have occurred, I will take the initiative to admit them and explain the situation to the team. | ○ | ○ | ○ | ○ | ○ |
| When making medical decisions, I will proactively evaluate the limitations of medical evidence. | ○ | ○ | ○ | ○ | ○ |

②Medical reflection habit

Please read the following statements carefully and choose the degree of agreement based on your true thoughts: [Matrix Single Choice]

|  | **Strongly Disagree** | **Disagree** | **Neutral** | **Agree** | **Strongly Agree** |
| --- | --- | --- | --- | --- | --- |
| After case discussions, I will review medical errors and record potential risk points. | ○ | ○ | ○ | ○ | ○ |
| When peers make medical errors, I analyze systemic factors rather than only blaming individuals. | ○ | ○ | ○ | ○ | ○ |
| I will discuss typical cases with the team and summarize effective problem-solving patterns. | ○ | ○ | ○ | ○ | ○ |
| For complex cases, I record the logic of medical decisions and mark uncertainties during diagnosis and treatment. | ○ | ○ | ○ | ○ | ○ |

③Medical reflection competency

Please read the following statements carefully and choose the degree of agreement based on your true thoughts: [Matrix Single Choice]

|  | **Strongly Disagree** | **Disagree** | **Neutral** | **Agree** | **Strongly Agree** |
| --- | --- | --- | --- | --- | --- |
| For errors in case analysis or diagnosis and treatment simulation, I can formulate specific improvement plans. | ○ | ○ | ○ | ○ | ○ |
| Under time pressure, I still adhere to standard medical procedures. | ○ | ○ | ○ | ○ | ○ |
| I believe my treatment plans for complex cases have clear logical evidence. | ○ | ○ | ○ | ○ | ○ |

1. **Ethical awareness**

Please read the following statements carefully and choose the degree of agreement based on your true thoughts: [Matrix Single Choice]

|  | **Strongly Disagree** | **Disagree** | **Neutral** | **Agree** | **Strongly Agree** |
| --- | --- | --- | --- | --- | --- |
| I can quickly identify ethical conflicts in medical situations. | ○ | ○ | ○ | ○ | ○ |
| I fully respect patients’ right to autonomy in medical decision-making. | ○ | ○ | ○ | ○ | ○ |
| I can support my medical choices by applying ethical principles such as beneficence, non-maleficence, and justice. | ○ | ○ | ○ | ○ | ○ |
| When facing medical ethical dilemmas, I will proactively seek discussions and assistance from third-party professionals such as ethics committees. | ○ | ○ | ○ | ○ | ○ |

**Note:** Only the questions are provided on the student questionnaire; no names of medical humanities dimensions are displayed.
