# Supplementary material for: Cultivating medical humanistic literacy through immersive case-based practical teaching in undergraduate pathophysiology education
Source: Front Med (Lausanne). 2026 Jun 24;13:1861607. doi: 10.3389/fmed.2026.1861607 (PMC13341589; doi:10.3389/fmed.2026.1861607)
Supplement: Supplementary file 2 [file Supplementary_file_2.docx]

**Sense of Gain Scale for the Immersive Case-based Practical Teaching Module**

Dear classmates,
This survey aims to understand your real experience and evaluation after participating in the immersive case-based practical teaching of the Pathophysiology course. The results will be used for the reform, optimization and improvement of course teaching and instructional design.

The questionnaire is anonymous, and all data are used only for overall statistical analysis. We strictly abide by research ethics to ensure the confidentiality of personal information.

There are no right or wrong answers. Please answer truthfully based on your actual situation. This survey is conducted solely for teaching research purposes and has no relation to your course scores or assessment.

Sincerely thank you for your support and cooperation!

The Pathophysiology Course Team

**After completing the immersive case-based practical teaching module, please answer the following questions based on your thoughts and feelings.[Matrix Single Choice]**

|  | Strongly disagree | Disagree | Neutral | Agree | Strongly agree |
| --- | --- | --- | --- | --- | --- |
| I believe the situational simulation of the immersive case-based practical teaching module allows me to truly experience the pressure of medical decision-making. | ○ | ○ | ○ | ○ | ○ |
| I believe immersive role-playing deepens my understanding of non-medical roles (e.g., patients, patients' family members). | ○ | ○ | ○ | ○ | ○ |
| I believe the interactive design of the immersive case-based practical teaching module prompts me to actively reflect on my own shortcomings. | ○ | ○ | ○ | ○ | ○ |
