# Supplementary material for: Cultivating medical humanistic literacy through immersive case-based practical teaching in undergraduate pathophysiology education
Source: Front Med (Lausanne). 2026 Jun 24;13:1861607. doi: 10.3389/fmed.2026.1861607 (PMC13341589; doi:10.3389/fmed.2026.1861607)
Supplement: Supplementary file 3 [file Supplementary_file_3.docx]

**Survey on extracurricular medical practice**

Dear classmates,

Hello! This survey aims to investigate the current status of participation and related willingness in extracurricular activities among medical students. The survey covers participation in social practice, voluntary service, internship/traineeship experience, academic lectures, innovation competitions and other activities. The results will be used for research on curriculum teaching reform and optimization of instructional design.

The questionnaire is anonymous, and all data are used only for overall statistical analysis. We strictly abide by research ethics to ensure the confidentiality of personal information.

There are no right or wrong answers. Please answer truthfully based on your actual situation. This survey is conducted solely for teaching research purposes and has no relation to your course scores or assessment.

Sincerely thank you for your support and cooperation!

The Pathophysiology Course Team

### **1. Which of the following types of extracurricular medical practice activities do you plan to participate in during the third semester and vacation of this academic year? [Multiple Choice]**

☐ Social practice

☐ Volunteer service

☐ Internship/Traineeship

☐ Academic lectures

☐ Scientific research

☐ Innovation competitions

☐ Overseas study tours

☐ Other _________________

**2. Which of the following types of extracurricular medical practice activities have you already participated in during the third semester and vacation of this academic year? [Multiple Choice]**

| ☐ Social practice _________________  ☐ Volunteer service _________________  ☐ Internship/Traineeship_________________  ☐ Academic lectures _________________  ☐ Scientific research _________________  ☐ Innovation competitions_________________  ☐ Overseas study tours _________________  ☐ Other _________________  ☐ Have not participated in any extracurricular activities |
| --- |

### **3. How frequently have you participated in extracurricular medical practice activities during the third semester and vacation of this academic year? [Single Choice]**

○ Once a week or more frequently

○ Once a month

○ Occasionally

○ Never

**4. Are you willing to participate in extracurricular medical practice activities in your free time in the following year? [Single Choice]**

|  | Strongly unwilling | Unwilling | Neutral | Willing | Strongly willing |
| --- | --- | --- | --- | --- | --- |
|  | ○ | ○ | ○ | ○ | ○ |

1. **What skills or experience do you most hope to gain from extracurricular medical practice activities? [Multiple Choice]**

☐ Leadership and decision-making ability

☐ Teamwork ability

☐ Communication ability

☐ Professional cognition

☐ Professional knowledge

☐ Other _________________

1. **Do you think extracurricular medical practice activities is helpful for improving medical humanism? [Single Choice]**

|  | No help at all | Little help | Neutral | Much help | Great help |
| --- | --- | --- | --- | --- | --- |
|  | ○ | ○ | ○ | ○ | ○ |
